# Construction and description of a constitutive plipastatin mono-producing *Bacillus subtilis*

**DOI:** 10.1186/s12934-020-01468-0

**Published:** 2020-11-10

**Authors:** Maliheh Vahidinasab, Lars Lilge, Aline Reinfurt, Jens Pfannstiel, Marius Henkel, Kambiz Morabbi Heravi, Rudolf Hausmann

**Affiliations:** 1 Institute of Food Science and Biotechnology, Department of Bioprocess Engineering (150K), University of Hohenheim, Fruwirthstraße 12, 70599 Stuttgart, Germany; 2 Core Facility Hohenheim, Mass Spectrometry Unit, University of Hohenheim, August-von-Hartmann-Str. 3, 70599 Stuttgart, Germany

**Keywords:** *Bacillus subtilis*, Lipopeptide, Surfactin, Fengycin, Biosurfactant, Promoter exchange, Strain engineering, Bottlenecks, Ornithine, Fungicide

## Abstract

**Background:**

Plipastatin is a potent *Bacillus* antimicrobial lipopeptide with the prospect to replace conventional antifungal chemicals for controlling plant pathogens. However, the application of this lipopeptide has so far been investigated in a few cases, principally because of the yield in low concentration and unknown regulation of biosynthesis pathways. *B. subtilis* synthesizes plipastatin by a non-ribosomal peptide synthetase encoded by the *ppsABCDE* operon. In this study, *B. subtilis* 3NA (a non-sporulation strain) was engineered to gain more insights about plipastatin mono-production.

**Results:**

The 4-phosphopantetheinyl transferase Sfp posttranslationally converts non-ribosomal peptide synthetases from inactive apoforms into their active holoforms. In case of 3NA strain, *sfp* gene is inactive. Accordingly, the first step was an integration of a repaired *sfp* version in 3NA to construct strain BMV9. Subsequently, plipastatin production was doubled after integration of a fully expressed *degQ* version from *B. subtilis* DSM10^T^ strain (strain BMV10), ensuring stimulation of DegU-P regulatory pathway that positively controls the *ppsABSDE* operon. Moreover, markerless substitution of the comparably weak native plipastatin promoter (P_*pps*_) against the strong constitutive promoter P_*veg*_ led to approximately fivefold enhancement of plipastatin production in BMV11 compared to BMV9. Intriguingly, combination of both repaired *degQ* expression and promoter exchange (P_*pps*_::P_*veg*_) did not increase the plipastatin yield. Afterwards, deletion of surfactin (*srfAA-AD*) operon by the retaining the regulatory *comS* which is located within *srfAB* and is involved in natural competence development, resulted in the loss of plipastatin production in BMV9 and significantly decreased the plipastatin production of BMV11. We also observed that supplementation of ornithine as a precursor for plipastatin formation caused higher production of plipastatin in mono-producer strains, albeit with a modified pattern of plipastatin composition.

**Conclusions:**

This study provides evidence that *degQ* stimulates the native plipastatin production. Moreover, a full plipastatin production requires surfactin synthetase or some of its components. Furthermore, as another conclusion of this study, results point towards ornithine provision being an indispensable constituent for a plipastatin mono-producer *B. subtilis* strain. Therefore, targeting the ornithine metabolic flux might be a promising strategy to further investigate and enhance plipastatin production by *B. subtilis* plipastatin mono-producer strains.

## Background

The preservation of food security is a global concern, especially with regard to increasing population. At the same time, there is a growing demand for organic agriculture products in both developed and developing countries. This high demand emphasizes the need for an effective, environmentally friendly alternative to chemical fertilizers, fungicides, insecticides, etc. *Bacillus* strains e.g. *B. subtilis*, *B. amyloliquefaciens* and *B. velezensis* are among the beneficial microorganisms known as effective cell factories that can produce many different secondary antimicrobial metabolites, including lipopeptides [1–3]. Plipastatin is a bioactive lipopeptide produced by *Bacillus subtilis.* In general, the main lipopeptides produced by *Bacillus* strains are classified in the three families of surfactin, iturin and fengycin [4, 5], including plipastatin as a member of the fengycin family [6]. Lipopeptides mostly display additional biological activities besides their amphiphilic properties. It is reported that the fengycin group exhibits a broad antagonistic effect on various soil-borne and post-harvest fungal phytopathogens, specifically on filamentous fungi [7, 8]. Besides antifungal activity, various authors [6–10] have frequently reported the antibacterial, antiviral and anticancer properties of fengycins. Additionally, these lipopeptides act also as the elicitor of induced systemic resistance in plants [11]. However, in contrast to the very well investigated applicability of surfactin, the application potential of fengycins have so far been investigated only in a few cases, principally based on poor bacterial productivity of fengycins and therefore, laborious and ineffective production approaches.

The structure of lipopeptides comprises a fatty acid connected to a peptide moiety [12]. The composition of amino acids in the circular peptide chain and length of the fatty acid residue provides a unique property for every lipopeptide [13]. Plipastatin consists of a decapeptide chain (L-Glu – D-Orn – L-Tyr – D-Thr – L-Glu – D-Ala – L-Pro –L-Gln – D-Tyr – L-Ile), 8 amino acids of which form a peptide ring and is linked to a 3-hydroxy fatty acid with 14–19 carbon atoms that may be saturated or unsaturated [14, 15]. Although, the structure of fengycin is almost identical to plipastatin, the enantiomers of the amino acids in positions 3 and 9 (Tyr3 and Tyr9) are present in the l- and d-form in plipastatin, respectively, while in fengycin, they are in the reverse configuration [6, 13, 16]. Plipastatin and other lipopeptides are formed by step-by-step reactions of specific non-ribosomal peptide synthetases (NRPSs) [17]. The lipopeptide biosynthesis depends on the 4-phosphopantetheinyl transferase Sfp which converts the inactive apoforms of NRPSs to the active holoforms [18]. In case of *B. subtilis* 168, the genome has two large operons of *srfAA-AD* and *ppsABCDE,* which encode the subunits of NRPSs for surfactin and plipastatin production, respectively. However, due to a single base duplication in *sfp* gene, *B. subtilis* 168 is incapable to synthesize these lipopeptides [19–21]. Nevertheless, after repairing the *sfp* mutation, its lipopeptide production is restored [22, 23].

Usually, *Bacillus* spp. encode for more than one lipopeptide synthetase, which are not synthesized simultaneously in the same growth phase showing the involvement of different regulators. More specifically, in the genome of bacteria that have the *ppsABCDE* operon, the *srfAA-AD* operon is always present as well [24, 25]. However, while surfactin is being produced in the late exponential phase, the biosynthesis of plipastatin has been characterized in the stationary phase [25–27]. Lipopeptide synthetases are usually regulated by complex regulatory networks in a growth-phase dependent manner. In the case of plipastatin, the *ppsABCDE* operon is repressed by AbrB, the transition state regulator, during exponential growth phase [28]. Moreover, expression of the pleiotropic regulator *degQ* gene has a positive effect on the synthesis of plipastatin showing the possible involvement of DegSU two-component system [20, 21, 29]. In *B. subtilis* 168, the –10 promoter region of *degQ* has a single base mutation that leads to low gene expression. Nevertheless, substitution of this mutation enables overexpression of the *degQ* gene and results in increased plipastatin biosynthesis [23, 30, 31].

In this study, we used *B. subtilis* 3NA as a model strain for genetic engineering of plipastatin production in order to construct a plipastatin mono-producer. This strain was previously described as a hybrid strain encoding genetic features from *B. subtilis* W23 and 168 [21]. In detail, the surfactin (*srfAA-AD*) operon was deleted in a way that their competence formation remained intact. Subsequently, the plipastatin was increased by promoter exchange and manipulation of the *degQ* expression. Finally, the effect of supplementing potentially critical amino acids on plipastatin formation was evaluated.

## Results

### Plipastatin production in different *B. subtilis* strains

**Fig. 1:**
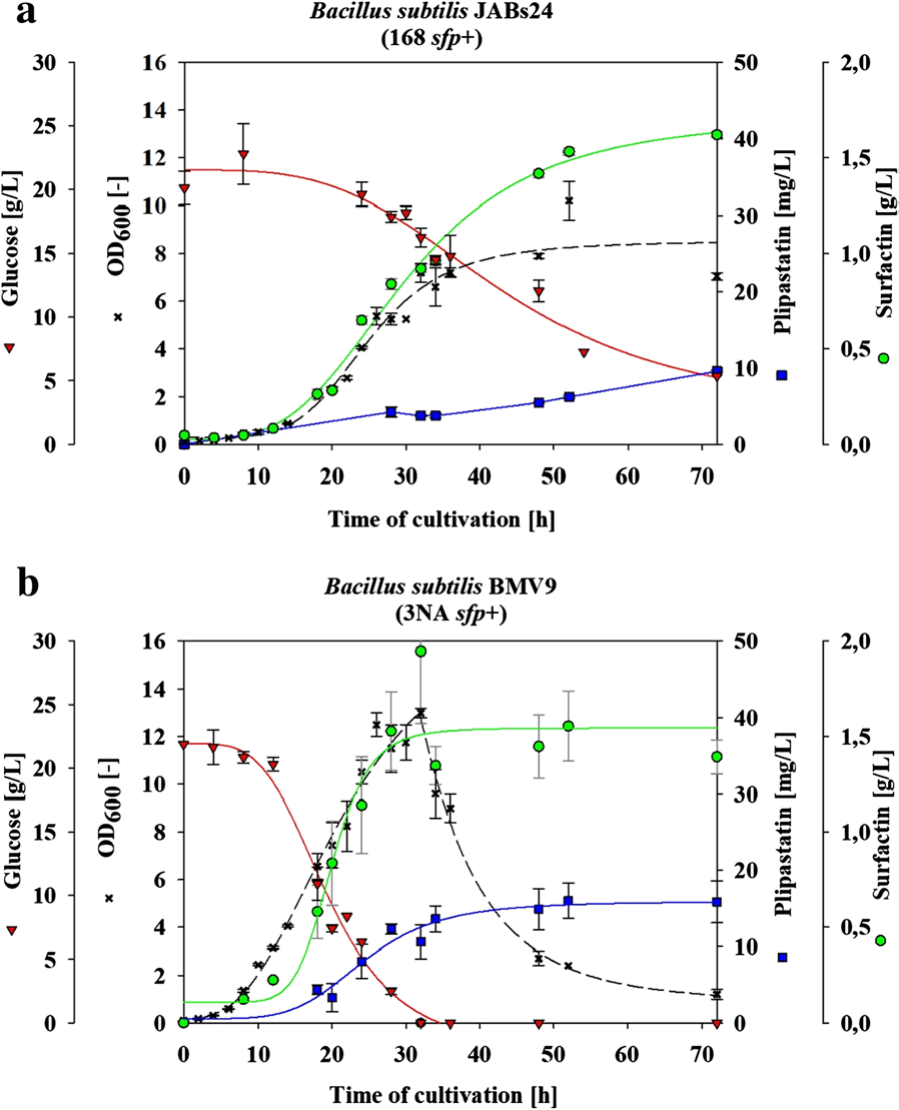
Comparison of cultivation parameters for **a** *B. subtilis* JABs24 (168 *sfp* +) and **b** *B. subtilis* BMV9 (3NA *sfp* +); The shake flask cultivations were performed in mineral salt medium containing 20 g/L glucose and 50 mM urea as carbon and nitrogen sources at 30 °C and 120 rpm. Besides optical density (OD_600_), glucose consumption, plipastatin and surfactin production were detected

To construct a plipastatin mono-producer, two *B. subtilis* strains were compared for their potential in biosynthesis of plipastatin. This included the derivatives of the well-known laboratory model strain *B. subtilis* 168 and strain 3NA. Plipastatin operon and its promoter region are identical in these two strains [21]. Nevertheless, strain 3NA is a sporulation-deficient strain caused by a frame shift mutation in *spo0A* which makes this strain suitable for fermentation [21]. These domesticated *B. subtilis* strains are known to have a mutation in their *sfp* gene disabling them to produce lipopeptides, such as surfactin and plipastatin [20, 21]. Therefore, the *sfp* + derivates of strain 168 (JABs24) and strain 3NA (BMV9) were used to ensure lipopeptide production. Both strains were cultivated in mineral salt medium. Figure 1 shows the lipopeptide production, glucose consumption and optical density (OD_600_) over 72 h of cultivation at 30 °C. The comparison of growth rates showed a faster cell growth of BMV9 strain compared to JABs24. However, optical density of BMV9 was drastically decreased without any stationary phase after glucose depletion. In contrast, JABs24 exhibited an entry into the stationary phase when 20% of glucose were metabolized. Both strains revealed surfactin production from the beginning of cultivation through to the early stage of stationary phase. Thus, surfactin quantities gradually increased until a peak was reached in the late exponential phase. In case of plipastatin, no detectable amounts could be measured up to the middle of exponential phase. The highest amount of plipastatin was produced by BMV9 in the stationary phase and was about 17 mg/L. Conversely, the production of plipastatin by JABs24 was lower than BMV9 (10 mg/L after 72 h). Based on these results strain BMV9 was considered for further strain engineering.

### Verification of different putative bottlenecks concerning plipastatin production

**Fig. 2:**
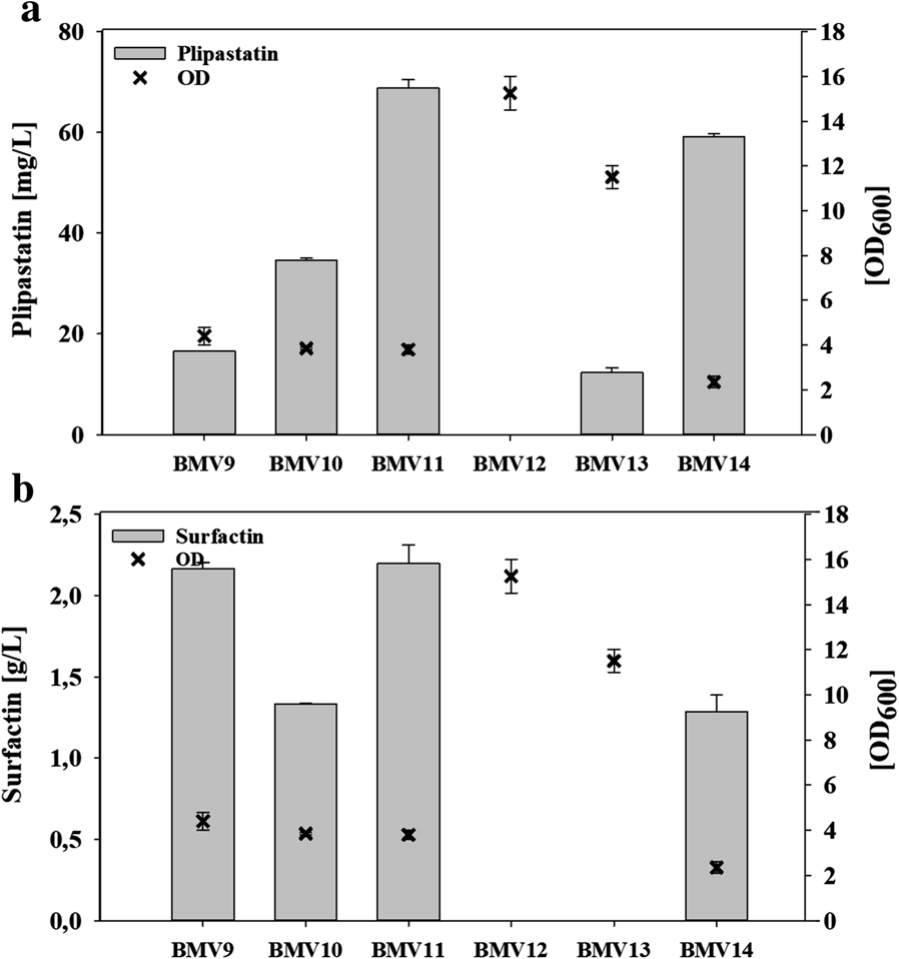
Overview about production of plipastatin (**a**) and surfactin (**b**) in respect to reached optical densities (OD_600_) of different engineered mutant strains after 48 h cultivation in mineral salt medium; BMV9, control strain (3NA *sfp* +); BMV10, repaired *degQ* expression; BMV11, promoter exchange of *pps* operon; BMV12, deletion of *srfA* operon; BMV13, combination of promoter exchange and *srfA* operon deletion; BMV14, combination of promoter exchange and repaired *degQ* expression

As mentioned before, *B. subtilis* 3NA is a hybrid strain that exhibits genetic features from both strains W23 and 168 [21]. Likewise, the *degQ* gene and upstream promoter region are identical to that of strain 168. Therefore, a single base mutation in the *degQ* promoter region drastically decreases the corresponding gene expression [32]. Previous studies demonstrated that this circumstance has a negative effect on plipastatin production [31]. We hypothesized that a repair of *degQ* expression combined with the deletion of the competitive surfactin operon as well as an exchange of a weak plipastatin promoter against a constitutively active promoter lead to a highly efficient plipastatin mono-producer strain. However, before constructing the mutant strain that exhibits these three characteristics, BMV9 strain (3NA *sfp* +) was used to construct BMV10, BMV11 and BMV12, showing repaired *degQ* expression, plipastatin promoter exchange and surfactin elimination, respectively. Based on the observation that plipastatin level reached a plateau after 40 h of cultivation (Fig. 1), the production outcomes of all following mutant strains were measured after 48 h of cultivation (Fig. 2).

As shown in Fig. 2, BMV10 which holds a repaired *degQ* gene, showed a twofold higher plipastatin production but a decrease in surfactin formation compared to the control strain (BMV9). Furthermore, the promoter exchange of native plipastatin promoter against the constitutive promoter region of the *veg* gene resulting in BMV11 strain, increased the plipastatin titer from 15 mg/L in control strain BMV9 to 70 mg/L. Interestingly, the surfactin formation was unaffected by the enhanced plipastatin production. In order to eliminate surfactin synthesis, the entire *srfAA-AD* operon was deleted and the regulatory *comS* gene which is co-expressed with *srfAA-AD* operon restored back to the genome with the native P_*srfA*_ promoter. Consequently, the strains BMV12 (Δ*srfAA*-*srfAD* with native P_*pps*_ promoter) and BMV13 (Δ*srfAA*-*srfAD* with P_*pps*_::P_*veg*_ promoter exchange) were constructed. It was observed that even though the deletion of the surfactin operon caused enhanced cell growth in both BMV12 and BMV13 strains, plipastatin production reduced (Fig. 2a). Plipastatin production was significantly decreased not only under native expression of plipastatin operon (BMV12) but also when P_*pps*_ was exchanged against constitutive P_*veg*_ promoter (BMV13).

In order to see the effect of combining the features of promoter exchange and repairing *degQ* gene expression, the strain BMV14 was constructed. Comparably to strain BMV9, the expression of *degQ* had a negative effect on surfactin production (Fig. 2b). Interestingly, no additive effect was observed on plipastatin production by BMV14 (Fig. 2a). In summary, we concluded that under the same conditions, BMV11 was able to produce the highest amounts of plipastatin compared to all other mutant strains constructed.

### Impact of amino acid supplementation on plipastatin production

To verify the impact of amino acid precursors on plipastatin and surfactin formation (Additional file 1), seven different amino acids including glutamic acid, glutamine, isoleucine, alanine, threonine, proline and ornithine with a concentration of 30 mM were additionally supplemented in mineral salt medium. BMV9 (3NA *sfp* +) and BMV12 (3NA *sfp* + plipastatin mono-producer) were selected to evaluate the produced plipastatin under control of native P_*pps*_ promoter, in the presence and absence of the surfactin operon.

**Fig. 3:**
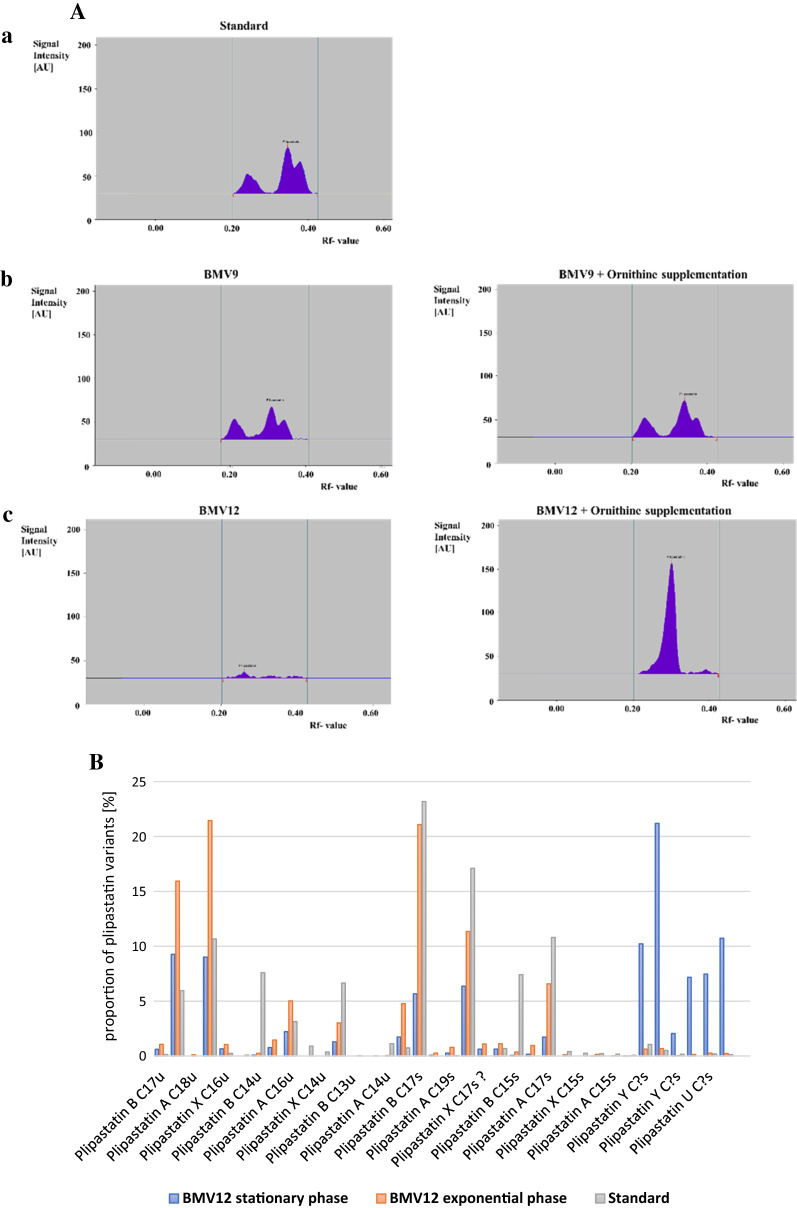
**A** Detected HPTLC chromatograms of the standard plipastatin in comparison with plipastatin produced by BMV9 (3NA *sfp* +) and BMV12 (3NA plipastatin mono-producer) with and without ornithine supplementation. Plipastatin standard produced by *B. subtilis* (Lipofabrik, France) [a], plipastatin produced by BMV9 strain after 48 h cultivation in mineral salt medium without any amino acid and with ornithine supplementation [b]; plipastatin produced by BMV12 strain after 48 h cultivation in mineral salt medium without amino acid and with ornithine supplementation [c]. **B** Comparison of plipastatin variants ratios in plipastatin standard and mono-producer BMV12 strain during exponential and stationary phase. The BMV12 was cultivated in mineral salt medium supplemented with 30 mM of ornithine. The samples were taken after 24 h (exponential phase) and 48 h (stationary phase). Peak areas were determined by extracted ion chromatograms for each plipastatin variant using their precise m/z values. Ratios of the peak areas of plipastatin variants were calculated within the standard and the two sample points

As it is shown in Table 1, except for ornithine, a decrease in plipastatin titer was observed when supplementing BMV9 strain with the other six amino acids. Interestingly, in the plipastatin mono-producer BMV12 strain, supplementation of the ornithine led to a detectable plipastatin titer. Neither in control cultures (without amino acid supplementation) nor in the presence of other amino acids, no detectable production was observed. Subsequently, it was observed that cultivation of BMV13 (constitutive plipastatin mono-producer) in supplemented medium with ornithine let to enhance in plipastatin titer (about 10%). Furthermore, it is noteworthy to mention that the observed plipastatin chromatogram on silica HPTLC plate of BMV12 (plipastatin mono-producer) in the presence of ornithine exhibited a modified pattern compared to the plipastatin standard (*B. subtilis* plipastatin/fengycin standard, Lipofabrik france) and parental BMV9 strain (Fig. 3A). Although the detected signals in the chromatogram showed similar Rf values compared to the standard, not all standard peaks could be observed. Therefore, HPLC–MS analysis was performed for identification of plipastatin variants or homologs.

**Table 1:** Plipastatin titers achieved of *B. subtilis* BMV9 (3NA *sfp* +) and *B. subtilis* BMV12 (3NA *sfp* + plipastatin mono-producer)

Supplementation	*B. subtilis* BMV9	*B. subtilis* BMV12
	Plipastatin (mg/L)	Plipastatin (mg/L)
Control	19.1 ± 0.1	n. d.*
Glutamic acid	7.8 ± 0.2	n. d
Glutamine	6.6 ± 0.9	n. d
Isoleucine	10.5 ± 2.6	n. d
Alanine	14.3 ± 0.9	n. d
Threonine	11.3 ± 0.8	n. d
Proline	14.4 ± 1.3	n. d
Ornithine	18.4 ± 2.4	6.3 ± 0.5

Plipastatin titers measured after 48 h cultivation in mineral salt medium supplemented with 30 mM of the indicated amino acids compared to control cultivations (without any amino acid supplementation).

*Not detectable

### Mass spectrometry analyses of plipastatin variants produced by BMV12 with supplementation of ornithine

Plipastatin produced by *B. subtilis* is a mixture of several homologs based on the length of fatty acid chain and variants within peptide moiety [33]. The patterns of the HPTLC chromatograms of produced plipastatin are comparable between the *B. subtilis* plipastatin standard and the sample extracts of the parental BMV9 strain. In both cases, plipastatin splits into several peaks. This might indicate that several plipastatin variants were produced (Fig. 3A [a, b]). Subsequently, plipastatin pattern of mono-producer strain BMV12 was analyzed by HPTLC and it appeared in one peak in the same Rf values (Fig. 3A [c]). Thus, it was assumed that deletion of *srfA* operon resulted in an accumulation of specific plipastatin variants whereas other subtypes were diminished. To get a perception about the different plipastatin variants present in *B. subtilis*, the high-purified plipastatin standard was analyzed by mass spectrometry (HPLC–ESI–MS). The results showed that a large variety of plipastatin subtypes with saturated and unsaturated fatty acid chains is present in the plipastatin standard (Additional file 2A). Mainly, A and B type plipastatin were detected in the LC–ESI–MS analysis, but also other plipastatin variants were observed. Some of them could be assigned to plipastatin C, D or S types [34] based on their diagnostic marker ions. In addition, other plipastatin variants were detected that were not described in previous studies in *B. subtilis* so far. Therefore, they were named in this study as W, U and Y. To get an overview about the differences in plipastatin variants produced by BMV12, shake flask cultivation was performed in mineral salt medium supplemented with ornithine. Samples were then harvested from the exponential and stationary phase (plipastatin HPTLC chromatograms were comparable in both growth phases) and also analyzed by LC–ESI–MS. Afterwards, the peak areas of identified plipastatin variants were used to determine ratios in between BMV12 samples and were compared to that of the plipastatin standard (Fig. 3B). Variants of the subtypes plipastatin A and plipastatin B were detected in both BMV12 samples and the standard, even though especially plipastatin B C_14_u (unsaturated fatty acid chain with 14 carbon atoms) and plipastatin B C_15_s (saturated fatty acid chain with 15 carbon atoms) showed higher relative abundance in the standard in contrast to BMV12 samples. In contrast, slightly higher abundances of plipastatin B C_16_u and plipastatin B C_18_s were detected in both samples of BMV12. Taken together, no major differences were detected between standard and BMV12 plipastatin extracts. Hence, the reason for the variation of the plipastatin pattern based on the deletion of surfactin operon could not be explained. Another interesting observation was the accumulation of different plipastatin variants (named in this study plipastatin Y, W and U) during the stationary phase. Due to the cultivation with mineral salt medium, a limitation of the amino acids during the stationary phase is very likely. Insofar, the accumulation of plipastatin Y, W and U could be due to the incorporation of different amino acids into the peptide residue of plipastatin.

## Discussion

In this study, *B*. *subtilis* 3NA was engineered to construct a plipastatin mono-producer strain and to gain more insights about plipastatin production. Previously, it was shown that DegQ positively regulates plipastatin production [30]. In fact, DegQ stimulates autophosphorylation of DegS sensor kinase resulting in enhanced phospho-transfer to DegU response regulator [29, 35]. As a result, the phosphorylated and activated DegU-P version causes higher expression of *ppsABCDE* operon and increases the plipastatin production [36]. In this study, repair of *degQ* expression (strain BMV10) ensured DegU-P activation, which doubled the production of plipastatin compared to parental BMV9 strain (3NA *sfp* +). In this context, previous results from Tsuge et al. [30] showed a tenfold higher plipastatin production when *ppsABCDE*, *degQ* and *sfp* were combined in a *B. subtilis* plasmid expression system. Furthermore, Wang et al [31]. described a decrease of plipastatin after in-frame mutagenesis of *degQ* in *B. subtilis *NCD-2. Afterwards, with respect to the relatively low expression level of *ppsABCDE* operon, an approximately fivefold higher plipastatin formation was achieved by the exchange of the native P_*pps*_ promoter against constitutive P_*veg*_ promoter (strain BMV11). Previous to this study, the effect of promoter exchange on the amount of plipastatin produced was reported only on a few cases. For instance, promoter exchange of native P_*pps*_ promoter against native P_*fen*_ from *B. subtilis* 21332 caused no plipastatin overproduction. In contrast, a tenfold higher plipastatin production was obtained when P_*fen*_ from strain BBG21 (a spontaneous mutant of *B. subtilis* ATCC 21332) was integrated [37]. In the following step, it was observed that the combination of both repair of *degQ* expression and the P_*pps*_ promoter exchange (BMV14), had no additional effect on the plipastatin titer compared to BMV11 (constitutive plipastatin producer). This is in contrast to the comparison of parental BMV9 and BMV10 when functional *degQ* expression increases plipastatin production about twofold. An explanation could be addressed by the DegQ mediated activation of DegU-P regulon, which causes in general the increase of secretory proteases [38, 39]. These proteases could target plipastatin for degradation. In sum, this negative effect could be more noticeable in a constitutive *pps* operon expression (P_*veg*_) which make a higher amount of DegQ not benefical.

Another aim of this study was to construct a strain that ensures constitutive plipastatin mono-production. In a recent work, it was shown that deletion of *srfAA* significantly reduced the plipastatin production and on the other hand deletion of *srfAC* showed no effect [25]. The authors argued that probably *srfAA* has a regulatory effect on plipastatin production. Furthermore, they also observed another significant decrease in plipastatin production when *pnp* gene, which is responsible for biosynthesis of multifunctional polynucleotide phosphorylase (PNPase) [40] was deleted. The regulation of PNPase on plipastatin biosynthesis can happen through effect on *comS* expression. Subsequently, it was hypothesized that *comS* expression in an unknown complex pathway positively regulates plipastatin formation. In this study, we have deleted the whole *srfAA-AD* operon and retained *comS* with the native P_*srfA*_ promoter (BMV12). In BMV12, even though *comS* was retained, plipastatin titer decreased to a non-detectable concentration confirming previously described study [25] that *srfAA* or in general, the expression of surfactin synthetase has a positive effect on plipastatin production. This observation was consistent after exchange of native P_*pps*_ against P_*veg*_ (BMV11 compared to BMV13). Likewise, in another study P_*pps*_ promoter was exchanged against a strong P_*amyQ*_ promoter from *Bacillus amyloliquefaciens* resulting in an increase in plipastatin production. However, after elimination of surfactin synthetase by deletion of *srfAB*, *srfAC* and *srfAD* genes, plipastatin production did not change [11]. Hence, in respect to our results and previously described studies [11, 25] we conclude that, subunits of the surfactin synthetase have different impacts on plipastatin production.

Another rational factor in increasing the plipastatin production is the presence of a sufficient quantity of precursors in the cultivation medium. Therefore, we have attempted to improve plipastatin production by supply of seven amino acids of the plipastatin peptide chain in the mineral salt medium. Previously, these seven amino acids as the only nitrogen source were used in the mineral salt medium and compared with other nitrogen sources such as urea and ammonium carbonate [41]. Accordingly, urea was introduced as the best nitrogen source and alanine, followed by glutamic acid, were the best sources of nitrogen among the other amino acids. In this study, approximately the same concentration of amino acids were added to the medium besides urea as the main nitrogen source. Since, surfactin synthetase, assumably has a regulatory effect on plipastatin production, the supplementation of amino acids was examined in two strains of BMV9 (3NA *sfp* +) and BMV12 (3NA *sfp* + plipastatin mono-producer). Among all the amino acids used, ornithine was the only amino acid induced a detectable plipastatin production in mono-producer (BMV12), while it has not been measured in the other cultivations of BMV12. Interestingly, in the strain BMV9, the supplementation of different amino acids had different effects on final plipastatin titer. As it was shown in Fig. 1, the optical density of BMV9 directly decreased after glucose consumption whereas the plipastatin titer remained stable over time. Therefore, a comparison of the plipastatin production per biomass after 48 h cultivation is not reasonable. Accordingly, the results show that expect for ornithine, the additional supplementation of amino acids reduced in general the plipastatin titer in BMV9. This can be explained due to (de-) activation of stringent response in *B. subtilis* which occurs in amino acid limitation. Activation of stringent response results in enhanced provision of branched-chain amino acids [42, 43]. Conversely, by addition of amino acids in the cultivation medium, the positive side effect of stringent response will be reduced. However, supplementation of ornithine in the medium had no negative effect and it was the only amino acid that enhanced the plipastatin produced by BMV12 (plipastatin mono-producer) to a detectable level. Therefore, it is concluded that ornithine is an indispensable constituent for a plipastatin mono-producer *B. subtilis* strain.

## Conclusions

This study provides evidence that *degQ* stimulates the native plipastatin production. A significant decrease in plipastatin productivity after deletion of the surfactin operon in a constitutive plipastatin producer strain suggested that full plipastatin production requires the surfactin synthetase or some of its components. Nevertheless, the impact of surfactin synthetase existence on plipastatin formation is still unknown. In order to construct a plipastatin mono-producer strain suitable for cultivation in large quantities in a bioreactor, understanding the mutual impact between surfactin and plipastatin syntheses might help to increase the final plipastatin production. Furthermore, as another conclusion of this study, results point towards ornithine provision being an indispensable constituent for a plipastatin mono-producer *B. subtilis* strain. Therefore, targeting the ornithine metabolic flux might be a promising strategy to further investigate and enhance plipastatin production by *B. subtilis* plipastatin mono-producer strains.

## Materials and methods

### Chemicals, materials and standard procedures

All chemicals were acquired from Carl Roth GmbH & Co. KG (Karlsruhe, Germany) if not, mentioned otherwise. Standard molecular techniques were carried out as described by Sambrook and Russell [44]. The desired DNA fragments were amplified in polymerase chain reactions using DNA Polymerase (Phusion High-Fidelity #M0530S, New England BioLabs, Frankfurt am Main, Germany). The PCRs were carried out on a PCR thermal cycler (prqSTAR 96X VWR GmbH, Darmstadt, Germany). Chromosomal DNA was purified with a ready to use kit (innuPREP Bacteria DNA Kit) and plasmid DNA was extracted with innuPREP Plasmid Mini Kit (Analytik Jena AG, Jena, Germany). After PCR reactions, amplified DNA fragments were extracted after agarose-based gel electrophoresis with QIAquick PCR & Gel Cleanup Kit, according to the manufactures’ instruction. Restriction enzymes and alkaline phosphatase (#M0290) was purchased from New England BioLabs (Frankfurt am Main, Germany) and T4 DNA ligase were purchased from Thermo Fisher Scientific (Karlsruhe, Germany). All ligation reactions were performed overnight at 4 °C. For better efficiency of ligation, a PEG 8000 solution was added. Oligonucleotides were synthesized by Eurofins MWG (Ebersberg, Germany).

### Strains, plasmids and transformation method

All strains and plasmids used in this study are shown in Table 2. Oligonucleotides used for construction of strains and plasmids are listed in Table 3. *Escherichia coli* JM109 was used for plasmid propagation and cloning. Transformation of *E. coli* strains were carried out according to the standard heat-shock method [45]. *B. subtilis* JABs32 strain, a *sfp* + derivate of *B. subtilis* 3NA, was used for mannose counterselection. Therefore, erythromycin resistance gene (*erm*) for *manPA* deletion was removed by the use of plasmid pJOE7644.2 resulting in BMV9 [46]. Strain BMV9 was used as parental strain for construction of further mutant strains. Transformation of natural competent *B. subtilis* strains was performed according to the “Paris method” [47]. Depending on the selection marker, the transformants were selected on LB agar supplemented with ampicillin (100 µg/mL), spectinomycin (100 µg/mL) or erythromycin (10 µg/mL for *E. coli* and 5 µg/mL for *B. subtilis*). All plates were incubated at 37 °C.

**Table 2:** Bacterial strains and plasmids used in this study

Strain or plasmid	Genotype or description		Reference
Strains			
*Escherichia coli*			
JM109	*mcrA recA1 supE44 endA1 hsdR17 (r*_*K*_^*–*^*m*_*K*_^+^*) gyrA96 relA1 thi ∆(lac-proAB) F'* [*traD36 proAB*^+^ *lacI*^*q*^* lacZ ∆M15*]		[48]
*Bacillus subtilis*			
JABs24	*B. subtilis* 168 Δ*manPA*; *trp* + ; *sfp* + ;		[49]
3NA	*spo0A3*;		[50]
JABs32	*spo0A3*; Δ*manPA::erm*; *sfp* + ;		J. Altenbuchner (unpublished)
BMV9	*spo0A3*; Δ*manPA*; *sfp* + ;		This study
BMV10	*spo0A3*; Δ*manPA*; *sfp* + ; Δ*amyE*:: *degQ* (from *B. subtilis* DSM10^T^)		This study
BMV11	*spo0A3*; Δ*manPA*; *sfp* + ; P_*pps*_-*ppsA-E*:: P_*veg*_–*ppsA-E*		This study
BMV12	*spo0A3*; Δ*manPA*; *sfp* + ; Δ*srfAA*-*AD*:: *comS-erm*		This study
BMV13	*spo0A3* Δ*manPA*; *sfp* + ; Δ*srfAA*-*AD*:: *comS -erm*; P_*pps*_-*ppsA-E*::P_*veg*_–*ppsABCDE*		This study
BMV14	*spo0A3*; Δ*manPA*; *sfp* + ; P_*pps*_-*ppsABCDE*::P_*veg*_–*ppsABCDE*; Δ*amyE*::*degQ* (from *B. subtilis* DSM10^T^)		This study
Plasmids			
pJOE6743.1	*ori*_pUC18_, *bla, spc*, *manP*, *ter*-*lacI*-*lacZα*-*ter*		[51]
pJOE7644.2	*ori*_pUC18_, *bla,*P_*manP*_-*manP*, *spc*, *manR*-*ctaO*		[46]
pJOE4786.1	*ori*_pUC18_, *bla*, *ter*-′*lacI*-*lacZα*-*ter*		[52]
pKAM312	*ori*_pBR322_, *rop*, *ermC*, *bla*, *amyE*′-[*ter*-P_*glcR*_-*lacZ*-*spcR*]- ′*amyE*		[46]
pMAV3	pJOE4786.1 containig P_*veg*_::P_*pps*_ exchange fragment (integrated by *Sma* I)	This study	
pMAV4	pJOE6743.1 containig P_*veg*_::P_*pps*_ exchange fragment (integrated by *Hind* III)	This study	
pMAV5	pKAM312 containing *degQ* (*B. subtilis* DSM10^T^) (integrated by *Hind* III)		This study

**Table 3:** Oligonucleotides used in this study

Name	Sequence 5′–3′	Purpose
s1009	CTGCCGTTATTCGCTGGATT	Integration of *degQ *gene (*B*. *subtilis *DSM10^T^) (+ 510 bp) in *amyE* locus underlined sequences highlight the *Nde* I and *EcoR *I restriction site
s1410	ATTATTAA**CATATG**CGGCGTACCTCATACGGATACAC	
s1409	ATTATTAA**GAATTC**CTCCTTGATCCGGACAGAATC	
s1010	AGAGAACCGCTTAAGCCCGA	
s1221	GGAAAGTGAAAAAAGGAGAAGG	Construction of P_*veg*_::P_*pps*_ promoter exchange
s1222	CCTATGCAGGTTTTCAACTGTTATTGATTTGCCAAAATGACAG	
s1223	CAGTTGAAAACCTGCATAGG	
s1224	TGCATCCACCTCACTACAT	
s1225	ATGTAGTGAGGTGGATGCATTGAGCGAACATACTTATTCTTTAAC	
s1226	CATTTAAAGAGATTCCATCCATTATGATATG	
s1162	CATGATTTTCAGGTCTGCAAGAAC	Construction of *srfAA-AD*:: *comS*-*erm*
s1163	GTTCAAACGTCTGCTCCTCCTTAATCTTTATAAGCAGTGAACATGTGC	
s1164	AGGAGGAGCAGACGTTTGAAC	
s1165	CTTCTCCCTCCAGCAGAAGTAC	
s1166	CTTCTGCTGGAGGGAGAAGTAGGTATAAATTTAAC-GATCACTCATCATGTTC	
s1167	GACCGATAGATTTTGAATTTAGGTGTC	
s1168	CACCTAAATTCAAAATCTATCGGTCGAATGCCAAT-TTCTGCATGGTATAATAG	
s1169	GGCAACCTGATGGATAAAGAAATTG	

### Construction of plasmids for strain engineering

For markerless promoter exchange, LFH-PCR method was used [53]. Accordingly, upstream and downstream wild-type sequence of *pps* promoter region was fused with *veg* promoter. After ligation into *Sma* I digested pJOE4786.1 resulting in pMAV3, target sequence was isolated by *Hind* III digestion and was subsequently integrated into pJOE6743.1 (results in pMAV4). Afterwards, plasmid pMAV4 was transformed into strain BMV9 followed by the protocol described before [51].

For the integration of the *degQ* gene including promoter region (+ 510 bp) and terminator structure from *B. subtilis* DSM10^T^, the primer s1410 and s1409, containing *Nde* I and *EcoR* I restriction sites, were used. After restriction digestion, the *degQ* fragment was ligated into pKAM312 [46] resulting in pMAV5. After transformation of pMAV5 into parental strain BMV9 and other mutant strains, transformants were selected on LB agar plates containing spectinomycin. To ensure the correctness of plasmids and mutant strains, all constructs were confirmed by sequencing (Eurofins Genomics Germany GmbH, Ebersberg, Germany).

### Deletion of surfactin operon and retain of *comS* gene

**Fig. 4:**
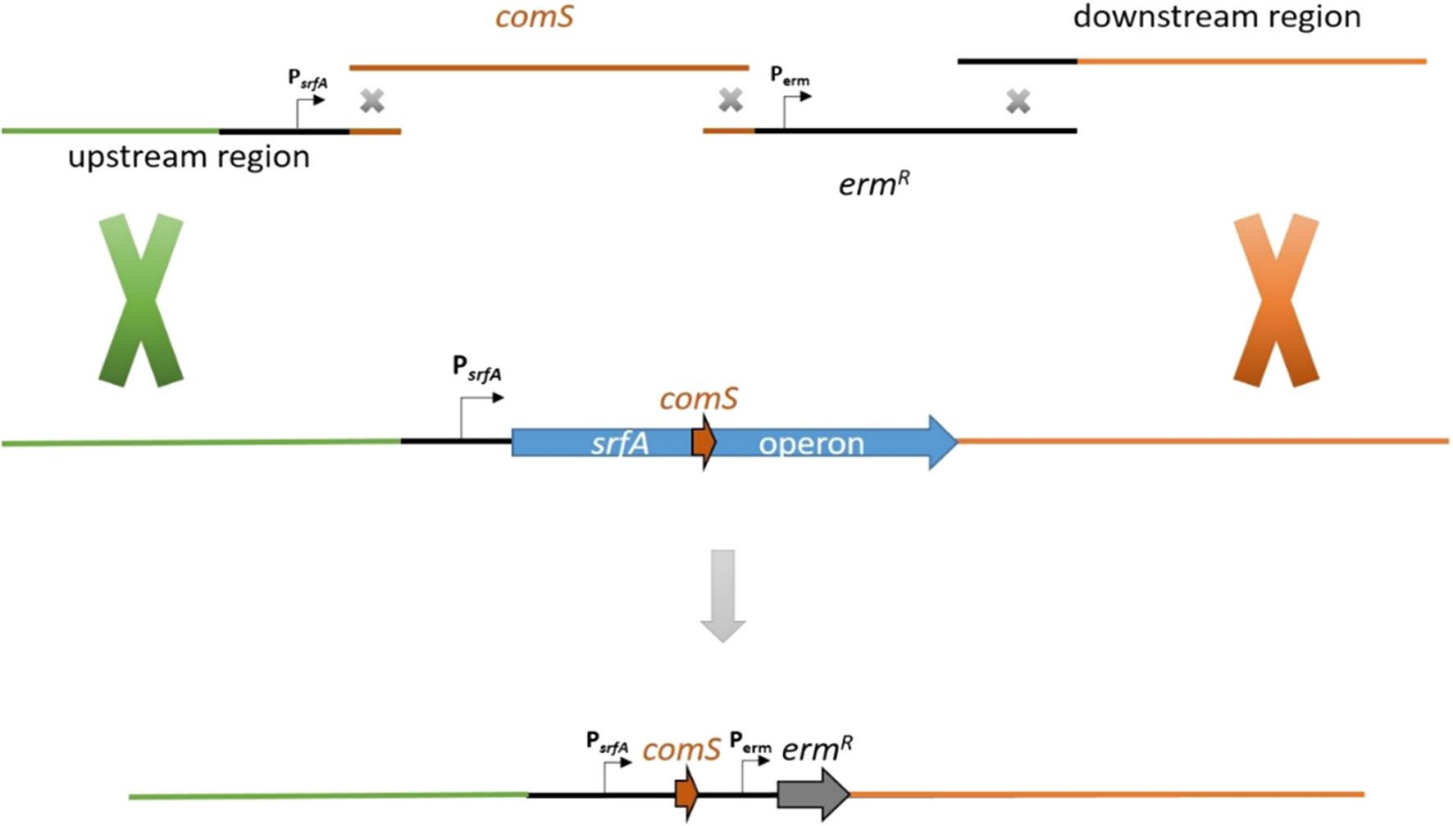
Schematic illustration of the construction of PCR fragment in order to delete surfactin operon and retain back *comS* with the native P_*srfA*_ promoter using LFH-PCR method

The principle of LFH-PCR was utilized to design a DNA fragment for deletion of *srfA* operon and simultaneously retain of *comS* gene. A fusion of upstream region of *srfA* operon with *comS* gene ensured a wild-type expression. For a simple strain selection, *comS* gene was additionally linked with erythromycin resistance cassette (*erm*) of pKAM312. An uncoupled *erm* gene expression was ensured by maintaining the natural P_*erm*_ promoter region from pKAM312. Figure 4 illustrates the described strategy.

### Cultivation in mineral salt medium

The mineral salt medium used was based on the fermentation medium of Willenbacher et al. [54] with slight changes. The composition of the final medium was: 20 g/L glucose, 4.0 × 10^−6^ M Na_2_EDTA × 2 H_2_O, 7.0 × 10^−6^ M CaCl_2_, 4.0 × 10^−6^ M FeSO_4_ × 7 H_2_O, 1.0 × 10^−6^ M MnSO_4_ × H_2_O, 50 mM Urea, 0.03 M KH_2_PO_4_, 0.04 M Na_2_HPO_4_ × 2 H_2_O and 8.0 × 10^−4^ M MgSO_4_ × 7 H_2_O.

For the first preculture, 10 mL LB medium (10 g/L tryptone, 5 g/L NaCl, 5 g/L yeast extract) was inoculated with 10 μL glycerol stock solution in a 100 mL baffled shake flask. After 8 h of cultivation, the cells were transferred to 10 mL mineral salt medium with an initial OD_600_ of 0.1 as a second preculture. This preculture was incubated overnight and after reaching an OD_600_ between 2–4 the main culture was inoculated. The main cultivations took place in 1 L Erlenmeyer baffled flasks with the final volume of 100 mL and initial OD_600_ of 0.1. All cultivation had three biological replicates and were performed at 30 °C and 120 rpm in an incubation shaker (Innova 44®R, Eppendorf AG, Hamburg, Germany).

Additionally, the influence of potentially critical amino acids including glutamic acid, glutamine, isoleucine, alanine, proline and ornithine on plipastatin production was tested using mineral salt medium complemented with 30 mM of each amino acids.

### Extraction of lipopeptides and HPTLC analysis

The cell-free supernatants were obtained by centrifugation at 4700 rpm and 4 °C and were used for extraction of lipopeptides following the method described before with slight changes [55]. In detail, 2 mL of cell-free supernatant was mixed 3 times with 1 mL 1-butanol 95% (*v/v*) by vortexing for 1 min, followed by 5 min centrifugation at 3000 rpm to separate organic phase. After complete evaporation of butanol phases (RVC2-25 Cdplus, Martin Christ Gefriertrocknungsanlagen GmbH, Osterode am Harz, Germany) at 10 mbar and 60 °C, the remaining residues were dissolved in 2 mL methanol. To quantify surfactin and plipastatin production, these methanolic fractions were separated by HPTLC (CAMAG, Muttenz, Switzerland) as described previously [56].

### Structural analysis of plipastatin variants by Mass spectrometry

LC–MS analysis of plipastatin was performed on a 1290 UHPLC system (Agilent, Waldbronn, Germany) coupled to a Q-Exactive Plus Orbitrap mass spectrometer equipped with a heated electrospray ionization (HESI) source (Thermo Fisher Scientific, Bremen, Germany). Analyte separation was achieved by a Waters ACQUITY CSH C18 column (1.7 μm, 2.1 μm × 150 mm). The column temperature was maintained at 40 °C. Samples were dissolved in methanol and 5 µl of each sample was injected. Mobile phase A was 0.1% formic acid in water (v/v), and mobile phase B 0.1% formic acid in acetonitrile (v/v). A constant flow rate of 0.3 mL/min was used and the gradient elution was performed as follows: 0 – 15% B from 0 to 15 min, 15–75% B from 15 to 29 min, 75–100% B from 29 to 32 min, isocratic at 100% B from 32 to 36 min, the system was returned to initial conditions from 100% B to 0% B from 36 to 37 min. The HESI source was operated both in positive and negative mode, with a capillary voltage of 3.90 kV and an ion transfer capillary temperature of 350 °C. The sweep gas and auxiliary pressure rates were set to 35 and 10, respectively. The S-Lens RF level was 50%, and the auxiliary gas heater temperature was 150 °C. The temperature of ion transfer capillary, spray voltage, sheath gas flow rate, auxiliary gas flow rate and S-lens RF level were set to 325 °C, 3.5 kV, 60, 30 and 55, respectively. The Q-Exactive Plus mass spectrometer was calibrated externally in positive and negative ion mode using the manufacturer’s calibration solutions (Pierce/Thermo Fisher, Germany). Mass spectra were acquired in MS mode within the mass range of 600–1800 m/z at a resolution of 70,000 FWHM using an Automatic Gain Control (AGC) target of 1.0 × 10^6^ of and 100 ms maximum ion injection time. Data dependent MS/MS spectra in a mass range of 200–2000 m/z were generated for the five most abundant precursor ions with a resolution of 17,500 FWHM using an Automatic Gain Control (AGC) target of 5.0 × 10^4^ of and 64 ms maximum ion injection time and a stepped collision energy of 20, 60 and 150. Xcalibur™ software version 4.0.27 (Thermo Fisher Scientific, San Jose, USA) was used for data acquisition and data analysis.

## Supplementary information

**Additional file 1.** Overview about production of surfactin and corresponding optical densities (OD_600_) of *B. subtilis* BMV9 (3NA *sfp* +) after 48 h cultivation in mineral salt medium supplemented with 30 mM of different amino acids.

**Additional file 2.** A: Plipastatin variants detected by MS analysis in *B. subtilis* standard. B: Detailed mass spectrometry data of extracted-ion chromatograms regarding to plipastatin standard produced by *B. subtilis* and the sample extracts of BMV12 strain from exponential phase and stationary phase.

## Data Availability

All raw data and biological material are saved in the institute of Food Science and Biotechnology, Department of Bioprocess Engineering (150 k), University of Hohenheim, Fruwirthstraße 12, Stuttgart 70599, Germany. In case of requirement, please contact the corresponding author for any detailed question.
