# Supplementary material for: Construction and description of a constitutive plipastatin mono-producing Bacillus subtilis
Source: Microb Cell Fact. 2020 Nov 10;19:205. doi: 10.1186/s12934-020-01468-0 (PMC7654001; doi:10.1186/s12934-020-01468-0)

**Supplementary Data_additional file 1.**

**Overview about production of surfactin and corresponding optical densities (OD_600_) of *B. subtilis* BMV9 (3NA *sfp*+) after 48 h cultivation in mineral salt medium supplemented with 30 mM of different amino acids.**
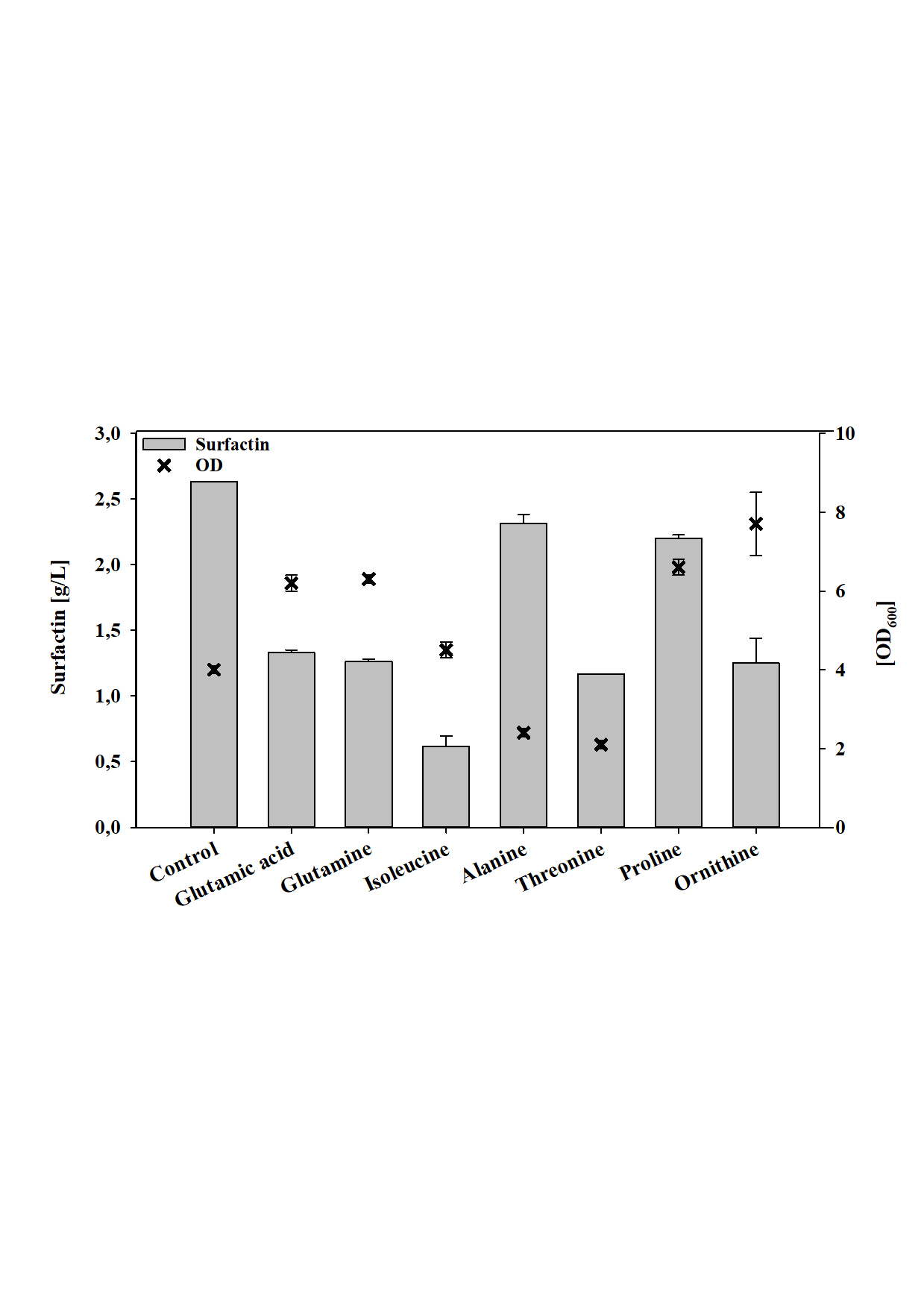

Supplement: Supplementary file 1 — Additional file 1. Overview about production of surfactin and corresponding optical densities (OD600) of B. subtilis BMV9 (3NA sfp +) after 48 h cultivation in mineral salt medium supplemented with 30 mM of different amino acids. [file 12934_2020_1468_MOESM1_ESM.docx]
