# Supplementary material for: Construction and description of a constitutive plipastatin mono-producing Bacillus subtilis
Source: Microb Cell Fact. 2020 Nov 10;19:205. doi: 10.1186/s12934-020-01468-0 (PMC7654001; doi:10.1186/s12934-020-01468-0)
Supplement: Supplementary file 2 — Additional file 2. A: Plipastatin variants detected by MS analysis in B. subtilis standard. B: Detailed mass spectrometry data of extracted-ion chromatograms regarding to plipastatin standard produced by B. subtilis and the sample extracts of BMV12 strain from exponential phase and stationary phase. [file 12934_2020_1468_MOESM2_ESM.docx]

**Supplementary Data_ additional file 2.**

**A: Plipastatin variants detected by MS analysis in *B. subtilis* standard (Lipofabrik, france).** Precursor ions (M+H)^+^, retention time, diagnostic fragment ions and corresponding Plipastatin homologue are shown.

| **m/z (M+H)^+^** | **Retention time (min)** | **Diagnostic ions** | **Plipastatin homologue** |
| --- | --- | --- | --- |
| **Plipastatin variants with unsaturated fatty acid chain** | | | |
| 1503,8759 | 26,34 | 994/1108 | Plipastatin B C_17_ |
| 1489,8545 | 25,66 | 994/1108 | Plipastatin B C_16_ |
| 1475,8384 | 25,24 | 994/1108 | Plipastatin B C_15_ |
| 1475,8384 | 25,58 | 980/1094 | Plipastatin X C_16_ |
| 1475,8384 | 25,97 | 966/1080 | Plipastatin A C_17_ |
| 1461,8229 | 24,60 | 994/1108 | Plipastatin B C_14_ |
| 1461,8229 | 25,28 | 966/1080 | Plipastatin A C_16_ |
| 1447,8053 | 24,22 | 994/1108 | Plipastatin B C_13_ |
| 1447,8053 | 24,51 | 980/1094 | Plipastatin X C_14_ |
| 1447,8053 | 24,85 | 966/1080 | Plipastatin A C_15_ |
| 1433,7936 | 23,62 | 994/1108 | Plipastatin B C_13_ |
| 1433,7936 | 23,90 | 980/1094 | Plipastatin X C_14_ |
| 1433,7936 | 24,25 | 966/1080 | Plipastatin A C_14_ |
| **Plipastatin variants with saturated fatty acid chain** | | | |
| 1519,8682 | 25,49 | 994/1108 | Plipastatin B C_18_ |
| 1505,8489 | 24,82 | 994/1108 | Plipastatin B C_17_ |
| 1505,8489 | 25,49 | 966/1080 | Plipastatin A C_19_ |
| 1491,8314 | 24,40 | 994/1108 | Plipastatin B C_16_ |
| 1491,8314 | 25,05 | 966/1080 | Plipastatin A C_18_ |
| 1477,8169 | 23,83 | 994/1108 | Plipastatin B C_15_ |
| 1477,8169 | 24,40 | 966/1080 | Plipastatin A C_17_ |
| 1463,8021 | 23,42 | 994/1108 | Plipastatin B C_14_ |
| 1463,8021 | 23,68 | 980/1094 | Plipastatin X C_15_ |
| 1463,8021 | 24,11 | 966/1080 | Plipastatin A C_16_ |
| 1449,7896 | 23,42 | 966/1080 | Plipastatin A C_15_ |
| 1435,7753 | 22,99 | 966/1080 | Plipastatin A C_14_ |
| **Unknown plipastatin variants with saturated fatty acid chain** | | | |
| 1523,8621 | 23,97 | 1012/1126 | Plipastatin Y C? |
| 1509,8499 | 23,56 | 1012/1126 | Plipastatin Y C? |
| 1495,8342 | 22,97 | 1012/1126 | Plipastatin Y C? |
| 1495,8342 | 23,56 | 972 and/or 1112 | Plipastatin U C? |
| 1481,818 | 23,16 | 984/1098 | Plipastatin W C? |

Detailed data of extracted-ion chromatograms of plipastatin standard produced by *B. subtilis* (Lipofabrik, France) and the extracts of plipastatin mono-producer BMV12 strain in exponential phase and in stationary phase. Strain BMV12 was cultivated in mineral salt medium supplemented with 30 mM ornithine.

Plipastatin A C14s

Plipastatin A C15s

Plipastatin X C15s

Plipastatin A C16s

Plipastatin B C15s

Plipastatin A C17s

Plipastatin B C15s

Fengycin A C18s

Plipastatin B C16s

Fengycin A C19s

Plipastatin B C17s

Plipastatin B C18s

**Extracted-ion chromatogram of Standard:** Plipastatin A and B with saturated fatty acid chains

Unknown Plipastatin or substance
with almost identical m/z

Plipastatin

Plipastatin B C16s

Plipastatin A C14s

Plipastatin A C15s

Plipastatin B C14s

Plipastatin A C16s

Plipastatin B C15s

Plipastatin A C17s

Plipastatin A C19s

Plipastatin B C17s

Plipastatin B C18s

**Extracted-ion chromatogram of BMV12 in exponential phase:** Plipastatin A and B with saturated fatty acid chains

m/z value not found, may be below detection limit

Unknown Plipastatin or
substance with almost identical m/z

Putative Plipastatin A C14s

Plipastatin A C16s

Plipastatin A C17s

Plipastatin B C16s

Plipastatin A C19s

Plipastatin B C17s

Plipastatin B C18s

**Extracted-ion chromatogram of BMV12 in stationary phase:** Plipastatin A and B with saturated fatty acid chains

Unknown Plipastatin

Unknown Plipastatin

Putative Plipastatin B C112u

Plipastatin A C14u

Plipastatin B C13u

Plipastatin A C15u

Plipastatin B C14u

Plipastatin A C16u

Plipastatin B C15u

Plipastatin B C16u

Plipastatin B C17u

**Extracted-ion chromatogram of Standard:** Plipastatin A and B with unsaturated fatty acid chains

Unknown Plipastatin or
substance with almost identical m/z

Unknown Plipastatin or
substance with almost identical m/z

Plipastatin A C14u

Plipastatin A C15u

Plipastatin B C14u

Plipastatin X C15u

Plipastatin A C16u

Plipastatin B C15u

Plipastatin B C16u

Plipastatin B C17u

**Extracted-ion chromatogram of BMV12 exponential in phase:** Plipastatin A and B with unsaturated fatty acid chains

Unknown Plipastatin or substance
with almost identical m/z

Putative Plipastatin X C16u

Unknown Plipastatin or substance
with almost identical m/z

Plipastatin A C15u

Plipastatin B C14u

Plipastatin X C15u

Plipastatin A C16u

Plipastatin B C15u

Plipastatin B C16u

Plipastatin B C17u

**Extracted-ion chromatogram of BMV12 in stationary phase:** Plipastatin A and B with unsaturated fatty acid chains

Unkown Plipastatin or substance*
with almost identical m/z

Plipastatin W C?s

Plipastatin W C?s

Plipastatin Y C?s

Plipastatin Y C?s

Plipastatin Y C?s

**Extracted-ion chromatogram of Standard:** Plipastatin Y and W with saturated fatty acid chains

Plipastatin W C?s

Plipastatin Y C?s

Plipastatin W C?s

Plipastatin U C?s

Plipastatin Y C?s

Plipastatin Y C?s

**Extracted-ion chromatogram of BMV12 in exponential phase:** Plipastatin Y and W with saturated fatty acid chains

Plipastatin W C?s

Plipastatin Y C?s

Plipastatin W C?s

Plipastatin U C?s

Plipastatin Y C?s

Plipastatin Y C?s

**Extracted-ion chromatogram of BMV12 in stationary phase:** Plipastatin Y and W with saturated fatty acid chains
